# An Accelerometer-Based Wearable Patch for Robust Respiratory Rate and Wheeze Detection Using Deep Learning

**DOI:** 10.3390/bios14030118

**Published:** 2024-02-22

**Authors:** Brian Sang, Haoran Wen, Gregory Junek, Wendy Neveu, Lorenzo Di Francesco, Farrokh Ayazi

**Affiliations:** 1 School of Electrical and Computer Engineering, Georgia Institute of Technology, Atlanta, GA 30332, USA; ayazi@gatech.edu; 2 StethX Microsystems Inc., Atlanta, GA 30308, USA; 3 Department of Medicine, Emory University School of Medicine, Atlanta, GA 30322, USA

**Keywords:** asthma, chronic obstructive pulmonary disease (COPD), accelerometer contact microphone, deep learning, remote patient monitoring (RPM), wheezing

## Abstract

Wheezing is a critical indicator of various respiratory conditions, including asthma and chronic obstructive pulmonary disease (COPD). Current diagnosis relies on subjective lung auscultation by physicians. Enabling this capability via a low-profile, objective wearable device for remote patient monitoring (RPM) could offer pre-emptive, accurate respiratory data to patients. With this goal as our aim, we used a low-profile accelerometer-based wearable system that utilizes deep learning to objectively detect wheezing along with respiration rate using a single sensor. The miniature patch consists of a sensitive wideband MEMS accelerometer and low-noise CMOS interface electronics on a small board, which was then placed on nine conventional lung auscultation sites on the patient’s chest walls to capture the pulmonary-induced vibrations (PIVs). A deep learning model was developed and compared with a deterministic time–frequency method to objectively detect wheezing in the PIV signals using data captured from 52 diverse patients with respiratory diseases. The wearable accelerometer patch, paired with the deep learning model, demonstrated high fidelity in capturing and detecting respiratory wheezes and patterns across diverse and pertinent settings. It achieved accuracy, sensitivity, and specificity of 95%, 96%, and 93%, respectively, with an AUC of 0.99 on the test set—outperforming the deterministic time–frequency approach. Furthermore, the accelerometer patch outperforms the digital stethoscopes in sound analysis while offering immunity to ambient sounds, which not only enhances data quality and performance for computational wheeze detection by a significant margin but also provides a robust sensor solution that can quantify respiration patterns simultaneously.

## 1. Introduction

Respiratory-related diseases, such as asthma and chronic obstructive pulmonary disease (COPD), are chronic conditions that can lead to many symptoms, such as difficulty in breathing, coughing, chest tightness, and respiratory wheezing. Asthma and COPD are the two leading chronic respiratory disorders, which each affect approximately 300 million people globally [1,2,3,4,5,6,7]. In addition, asthma is expected to affect 400 million people by 2025, and COPD is expected to be the global leading cause of death in 10 years, both due to increased exposure to environmental allergens and pollutants and sub-optimal primary care systems [6,8,9]. Globally, it has been estimated that asthma and COPD are underdiagnosed by 20–70% and 80%, respectively [10,11]. A major cause of underdiagnosis is lack of access to quality health care and poor self-management [12,13]. Addressing the issue involves improving access to quality healthcare for early detection and tracking the progression of asthma and COPD. This can be achieved through cost-effective and noninvasive remote patient monitoring (RPM), facilitating the identification of pre-symptomatic or subclinical respiratory changes [14,15,16,17]. RPM can be enabled by novel wearable patch solutions [18,19]. However, for accurate and comprehensive monitoring of respiratory health changes, it is essential to employ robust diagnostic patches. These robust wearable patches are needed to monitor and detect relevant respiratory changes that could signal physicians to provide more timely and effective healthcare to patients [20].

### 1.1. Lung Auscultation

Lung auscultation stands out as a clinically proven, non-invasive method for pulmonary diagnosis, highlighting its potential for pulmonary monitoring. For many years, physicians have used auscultation to subjectively listen to the acoustics of the lungs for adventitious lung sounds like wheezing, a key indicator of airway obstructive disorders such as asthma and COPD. Wheezing is due to air flowing through narrowed airways and other mechanical forces, which causes a high-pitched whistling sound emanating from the lungs that is transmitted to the patient’s chest walls [21,22,23]. The frequency of the whistling sound could also provide clinically relevant information about the respiratory status, more specifically, whether the patient has monophonic or polyphonic wheezing [24,25]. In addition, due to the possible focality of wheezing found in some acute and/or chronic respiratory disorders, such as COPD, and the relatively large size of the lungs, a comprehensive anatomically based examination of a patient’s lungs is needed to provide a more accurate clinical diagnosis [26]. In summary, wheeze characteristics, such as its frequency, presence during inspiration or expiration, and its lung anatomical location, can provide physicians with a more specific diagnostic framework for the presence of decompensated respiratory disorders like asthma and COPD, which can facilitate a more refined clinical treatment plan [25,27,28].

While lung auscultation remains a powerful diagnostic tool, even with advancements like digital stethoscopes being able to record chest sounds [29], barriers to widespread pulmonary remote patient monitoring use persist. Challenges include the lack of continuous monitoring via auscultation, the bulkiness of current digital stethoscopes, and the necessity for a trained medical professional to validate traditional and/or digital findings. [30,31]. In addition, stethoscopes are often used in uncontrolled environments and can pick up interfering ambient sounds (e.g., people talking, monitoring device alarms, etc.), which makes it challenging to distinguish sounds along with the source of those sounds [29,31,32,33].

To circumvent these problems, a promising, novel approach is to capture only the acoustic vibrations of the chest walls using a chip-scale wideband accelerometer, which acts as a contact microphone by measuring the vibrations of a surface that it is mounted on while also isolating from ambient sounds [29,30,34,35,36,37,38,39]. This is used in a miniature wearable patch and deploys objective and validated methods, like computation detection, to detect wheezing [32,40,41,42,43].

This study introduces robust computational methods for automated wheeze detection in patients, leveraging data from a sensitive wearable accelerometer patch capturing pulmonary-induced vibrations (PIVs). Benchmarking a deterministic time–frequency analysis and a deep learning model, these methods, when combined with the wearable accelerometer patch, offer a standardized approach to detect respiratory wheezing and monitor the pulmonary system while also not being sensitive to ambient sounds.

### 1.2. Accelerometer-Based Wearable Patch

**Figure 1:**
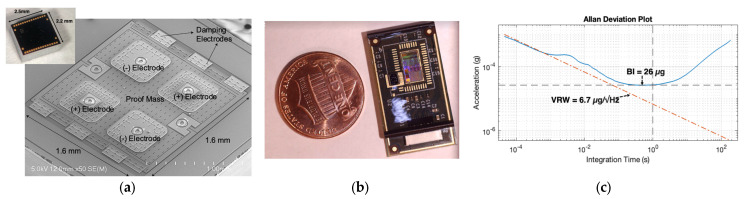
(**a**) The MEMS sensor is a wideband accelerometer with out-of-plane sensitivity and micro-g resolution made from a suspended silicon membrane operating in vacuum employing differential nano-gap capacitive transducers. (**b**) Accelerometer patch with compact size, which encompasses the CMOS ASIC. (**c**) Measured Allan Deviation of MEMS + ASIC exhibiting state-of-the-art noise performance of 6.7 µg/√Hz (20× lower noise than [34]).

**Figure 2:**
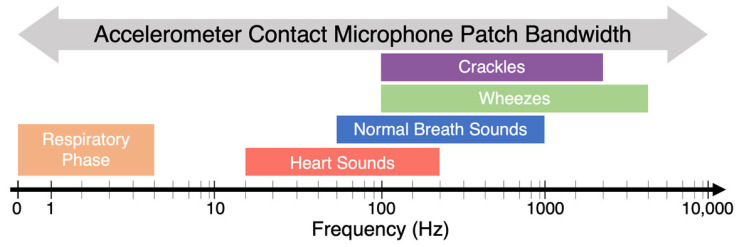
Accelerometer patch bandwidth, which encompasses the frequency of respiratory phase, heart sounds, normal breath sounds, wheezing, and crackles [21,37,42].

**Figure 3:**
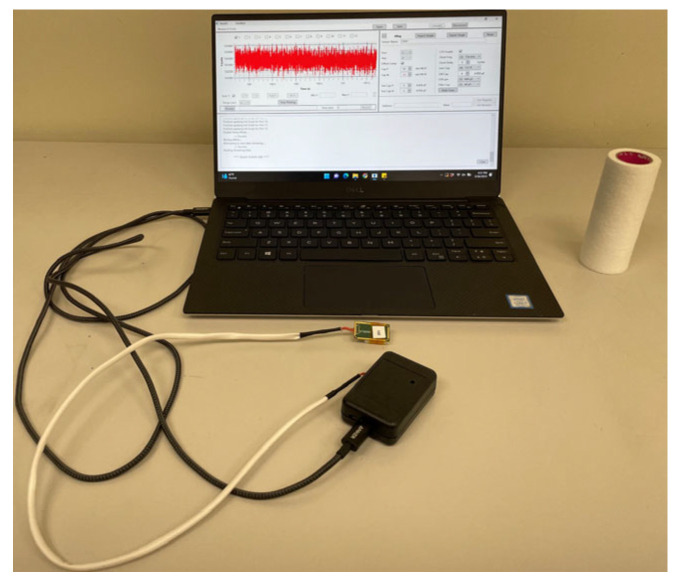
The accelerometer patch is attached to data-acquisition unit hub attached via USB C wire to the computer for data acquisition.

The sensor used in this work is a hermetically sealed micro-electromechanical system (MEMS) accelerometer with very high out-of-plane sensitivity, enabling micro-g/rtHz noise resolution in a wide dynamic range of ±6 g and a wide bandwidth of 10 kHz (Figure 1a). The device can act as a conventional DC accelerometer and a wideband vibrometer, i.e., a contact microphone, to enable the detection of low-frequency chest movements due to breathing as well as high-frequency vibrations on the chest due to PIVs, as shown in Figure 2. The accelerometer contact microphone (ACM) patch shown in Figure 1b is provided by StethX Microsystems, Atlanta, GA, USA, and can capture wideband PIVs from chest wall movements to 10 kHz due to its 20 kHz sampling rate without sensitivity to airborne sounds. The MEMS chip is interfaced with a custom high-bandwidth, low-noise CMOS (complementary metal-oxide semiconductor) ASIC (application-specific integrated circuit) for converting the MEMS capacitive output to a 24-bit digital signal, which can be manufactured together for a few USD. The velocity random walk (VRW), which is a representation of the thermal noise in the system, is measured at 6.7 µg/√Hz for the MEMS + ASIC configuration, as shown in Figure 1c, with 20× lower noise compared to the previous version, which used off-the-shelf data conditioning electronics [30]. The wearable sensor is placed on the chest wall via medical tape. The sensor is attached to a data-acquisition unit, programmed in C# with a custom lower-level library communication protocol, which interfaces with a computer to view the data through a GUI (graphical user interface), as shown in Figure 3 (all provided by StethX Microsystems).

## 2. Methodology

### 2.1. Participants

Lung sound recordings were taken from 52 patients admitted to Grady Memorial Hospital in Atlanta, GA, USA, in either an outpatient pulmonary asthma clinic or in the hospital setting (emergency room or general hospital bed) to provide a more representative sample pool and dataset of the use of auscultatory devices in real-world environment. The patients had varying demographics, such as age, height, weight, and body mass index, (BMI) as shown in Table 1 and Table 2. All the human subjects participated voluntarily with informed consent. In the outpatient asthma clinic, patient selection was guided by a diverse range of severity of asthma and lung sounds, whereas in the hospital, patients were chosen through blind referrals given to the physician for patients likely to exhibit adventitious lung sounds. The protocol was approved by Emory University and Georgia Institute of Technology Institutional Review Board (IRB #00105563). The process of data collection from patients was supervised by experienced and authorized physicians.

### 2.2. Study Protocol

**Figure 4:**
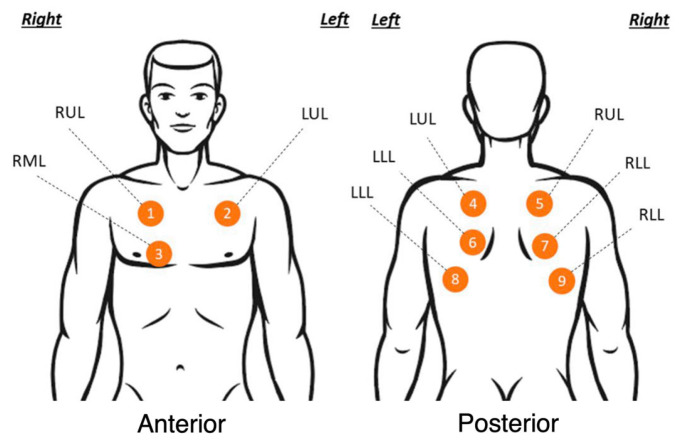
The sensor was typically placed on each of the nine auscultation sites used in common lung examination, which include the anterior and posterior of the chest. The site labels use a three-letter code: R represents right, M represents middle, L as the 1st letter represents left, L as the 2nd represents lower, and L as the last letter represents lobe.

**Figure 5:**
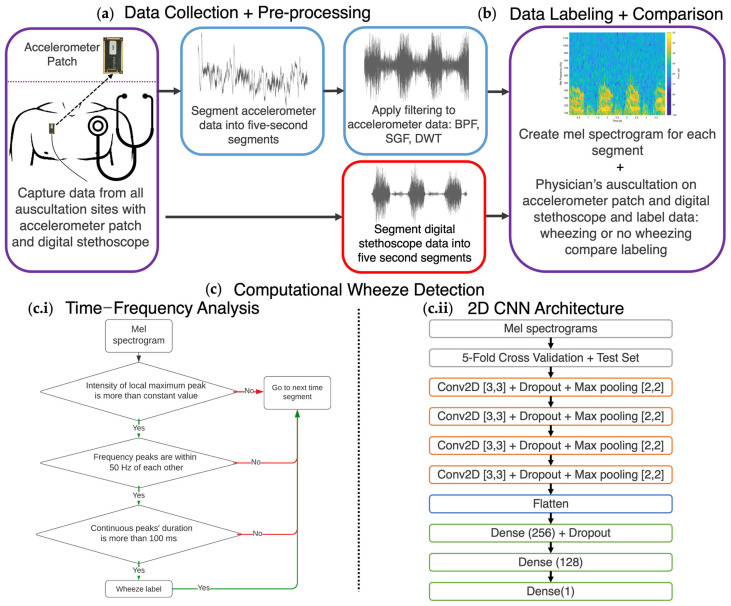
(**a**) Data were collected at auscultation sites by both the accelerometer patch, highlighted in red outline, and the digital stethoscope. For the accelerometer patch data, each recording was separated into five-second segments, and a bandpass filter (BPF), Savitzky–Golay filter (SGF) and discrete wavelet transform (DWT) were applied to the segment. The digital stethoscope recordings with a pulmonary filter were also segmented into 5-second bits with no further preprocessing. (**b**) Afterwards, mel spectrogram from the accelerometer patch and digital stethoscope segments were created via Fast Fourier Transform (FFT). In addition, both the accelerometer patch and digital stethoscope audios were analyzed while the accelerometer patch recording segments were played and labeled by physician. (**c.i**) Afterward, with the accelerometer patch/digital stethoscope mel spectrograms, a time–frequency approach was taken to detect if there was a wheeze and other characteristics of a wheeze. (**c.ii**) In addition, a deep learning approach with a 2D convolutional neural network was used with all the accelerometer patch/digital stethoscope mel spectrogram files with a 5-fold cross validation and test set to determine if a wheeze was present.

The accelerometer patch was placed on the patient’s chest walls and secured via 3M Medipore H-Soft Cloth Surgical Tape. Each patient was asked to sit or lie down and was then asked to take continuous deep breaths for 30 s intervals for each of the nine auscultation areas, as shown in Figure 4, while the accelerometer patch collected data. The accelerometer sensor board was connected via wire to a data-acquisition hub unit and then connected to a computer, as shown in Figure 3. A traditional stethoscope along with a digital stethoscope embedded in the same traditional stethoscope, specifically the Eko Core 3.0 (Berkeley, CA, USA) [44], was used by physicians, to which they applied direct pressure of the stethoscope diaphragm to the chest walls to listen and record lung sounds from the same auscultation areas. In the hospital/emergency room setting, the physician conducted their pulmonary examination with a traditional stethoscope, and then both the accelerometer patch and digital stethoscope were tested simultaneously in all auscultation sites close to each other from sites 1 to 9, as shown in Figure 5a. In the outpatient asthma clinical setting, the physician first conducted their traditional pulmonary examination with their stethoscope, provided auscultation notes, and then recorded respiratory sounds from the digital stethoscope on all or majority of auscultation sites. Some auscultation sites, specifically auscultation site 3, were not tested in outpatient asthma clinics due to clothing access restrictions. To address time constraints in the clinic, accelerometer patch data collection was conducted following the patient’s routine check-up. After testing in the hospital and clinical setting, the sound recordings of the accelerometer patch were played along with the gold standard—the digital stethoscope—based on auscultation sites to the physician, who then blindly labeled whether they heard wheezing or no wheezing, as shown in Figure 5b. Finally, the diagnosis of the patient’s clinical disease was provided by the physician after clinical evaluation and/or chart review, as shown in Table 2.

### 2.3. Signal Denoising and Analysis

With the accelerometer patch, the PIVs were captured, further processed, and denoised in MATLAB_R2021a, as shown in Figure 5a. Specifically, the PIVs were filtered with a band pass filter of 60 Hz to 2000 Hz, corresponding to the main lung sound frequencies and to remove the majority of heart sounds and motion artifacts from the signal, with a filter order of 10 [42]. The PIVs were then smoothened with a Savitzky–Golay Filter (SGF) with a window width of nine samples, as similarly used in digital stethoscope signal smoothening [45]. Afterward, the PIVs were denoised with Daubechies(db)3 Wavelet through discrete wavelet transform (DWT) to make the signal, when played, sound more similar to wheezes [46]. The data were then segmented into five-second segments and scaled from −1 to 1 for audible sound playback. For benchmarking the accelerometer patch with the Eko digital stethoscope, the Eko Core pulmonary filter was applied. Accelerometer patch, acting as a contact microphone, and digital stethoscope recordings were played to a physician to determine whether the audio contained wheezing or not. The accelerometer patch’s five-second clips were then labeled to be used later for computational wheeze detection. Since respiratory sounds have a wide range of frequencies with wheezes specifically ranging from 100 Hz–2 kHz [25,41], the accelerometer patch PIVs and the digital stethoscope recording were analyzed via mel spectrograms—a popular visual representation of the audio signals to showcase the frequency spectrum of how human perceive sounds—where the *x*-axis represents time (s), y-axis represents frequency (Hz), and the color scale represents power in sound decibel (dB), as shown in Figure 5b [41,47]. To create mel spectrograms, spectrograms from the scaled accelerometer data were first created via Fast Fourier Transform (FFT) with a Hann window length of 1024 samples (approximately 50 ms) with a 50% overlap, and the shown frequency range was set from 60 to 1200 Hz with 64 bands. Digital stethoscope spectrograms were also first created via FFT with a Hann window length of 256 samples (approximately 60 ms) with a 50% overlap, and the shown frequency range was set from 60 to 1200 Hz with 64 bands. The spectrogram parameters were chosen to reduce spectral leakage and time domain distortion. The spectrogram frequency axis, *y*-axis, was then converted into mel scale (m), a logarithmic scale correlated to how humans perceive pitch/frequency (f), as shown in Equation (1), to create mel spectrograms:

(1) $$m=2595\;\log_{10}\left(1+\frac{f}{700}\right)$$

To extract the chest wall movements for respiration rate and phase estimation from the accelerometer data, a low-pass filter of 5 Hz with a filter order of 10 was applied along with a moving average smoothing filter to show when these adventitious lung sounds occur based on the respiration phase, and mean centering was applied to show the overall amplitude range of the chest wall movements throughout the patient’s respiratory cycle. The accelerometer patch, based on an out-of-plane capacitive accelerometer, allows for extraction of near DC levels to measure chest wall movements directly linked to respiration phase. Since the patients were sitting upright and asked to take deep breaths, the upward ramps of amplitude plots indicate inspiration, while the downward ramps indicate expiration. All expiration is represented in the chest wall movement plots as shaded blue boxes. The accelerometer patch and digital stethoscope recordings with sound playback were then analyzed by physician and labeled to determine accuracy and agreement of both devices.

### 2.4. Wheeze Detection Time–Frequency Analysis

To determine the optimal objective wheeze-detection method, two automatic wheeze-detection methods were formulated: a deterministic time–frequency approach to detect wheezes via its features like frequency and sound decibel (dB), and a deep learning model, specifically a 2D convolutional neural network (CNN), to determine if wheezing is present in a specific recording segment from the accelerometer patch. For the deterministic approach, mel spectrograms showed the frequencies of sound, as shown in the flow chart in Figure 5c.i. Using the mel spectrograms, local maximum peaks could then be determined at each time point, and the maximum peaks were extracted if they were above the constant value of the mel spectrogram. These extracted local maximum peaks were also to be determined within a range of 50 Hz of the next time segment local maximum peaks, as a wheeze is defined as a constant sound with the same frequency range. Afterward, calculations were performed to determine if the local maximum peaks were to be continuous for greater than 100 ms to differentiate between other peaks, such as breath sounds or other adventitious lung sounds, and to confirm wheezing based on previously standardized computational wheeze characteristics [48]. Finally, the output was examined to determine if and when wheezing was present in the five-second segments. The accuracy rate was determined by comparing the outputs with the physician’s labeling using their traditional lung auscultation.

### 2.5. Classification of Wheezing via Convolutional Neural Network (CNN)

By using the 5 s segment mel spectrograms from 52 patients, a 2D CNN from TensorFlow GPU and Keras was created with a total of 1356 respiratory wheezing sounds and 1334 normal breath sounds mel spectrograms with no padding, as shown in the flow chart in Figure 5c.ii. The 2D CNN model was chosen for its low latency along with its high performance in speech detection. Initially, 20% of the data was randomly designated as test data, while the remaining data were used as training data. The training data were separated for a five-fold cross-validation method and were used to validate performance of hyperparameter optimization. Early stopping was utilized to monitor validation loss. No additional pre-processing of the data, such as data augmentation, was performed. This model utilized four filters of 32, 64, 128, and 256 with a kernel size of (3,3). Each of the four convolutional layers was followed by a dropout of 0.25 and then a max pooling 2D step with a filter size of (2,2) and rectified linear unit (ReLU) activation [49]. After all the convolutional layers, the data were then flattened. A dense linear layer with ReLU activation was applied, followed by a dropout of 0.5. Afterward, another dense linear layer followed. Finally, a dense output was created. The variables were optimized using the Adam optimization algorithm with a learning rate of 0.001. Accuracy, sensitivity, specificity, and the area under the curve (AUC) of the receiver operating characteristic (ROC) and Precision Recall (PR) were measured.

### 2.6. Digital Stethoscope Computational Wheeze Detection

In addition, the deterministic time–frequency analysis and the deep learning classifier wheeze detection methods were tested on the data captured from the digital stethoscope on the same patients to determine whether the accelerometer patch or digital stethoscope provided higher-quality data for computational wheeze-detection methods. The deterministic time–frequency method was unchanged. The wheeze digital stethoscope deep learning classifier retained the structure of the accelerometer patch deep learning model but included the mel spectrograms of 1210 wheezing sounds and 1402 normal breath sounds from the digital stethoscope.

## 3. Results

### 3.1. Patient Clinical Characteristics

We studied 52 patients with a diverse demographic and a wide variety of clinical characteristics examined in either an outpatient asthma clinic or the hospital setting, as shown in Table 1 and Table 2. With the accelerometer patch placed on a patient’s chest wall auscultation sites, the patient’s PIVs can be captured along with their synchronous chest wall movements that correspond to their respiratory phase, as outlined in the Methods. Combining the mel spectrogram with the synchronized time domain respiratory phase enables a more comprehensive analysis for physicians, as knowing if the wheeze is during expiration or inspiration can be clinically relevant [22].

The recordings of the accelerometer patch and digital stethoscope had great alignment via sound playback to a physician. In accordance with what the physician heard from the digital stethoscope recordings, the presence of a wheeze matched with the physician’s traditional stethoscope auscultation findings. The physician also determined that the accelerometer patch and digital stethoscope recordings were found to align in their classification of wheezing and non-wheezing sounds, as the agreement was 97.51% accurate with a 95.14% sensitivity and 98.56% specificity. The majority of these inconsistencies were from testing in outpatient clinics when the accelerometer patch and digital stethoscope were tested separately, as time and increased repetition of deep breaths along with coughs can cause the wheezing to move to different locations in the airways, flare, disappear.

After collecting data from 52 patients from 8 to 9 auscultation sites for 30 s each, a total of 1356 wheezing and 1334 non-wheezing five-second segments were captured and utilized in the computational wheeze detections. Out of those 52 patients, 16 of the patients had wheezing respiratory sounds, 20 of the patients had normal breath sounds, and 16 of the patients had both.

### 3.2. Comparing Time–Frequency Wheeze Detecting with Deep Learning Wheeze Classifier Using Accelerometer Mel Spectrograms

After the physician labeled the mel spectrograms of each recording segment, the accelerometer patch time–frequency wheeze detection and 2D CNN wheeze detection were benchmarked with each other as followed in Figure 5c.i and Figure 5c.ii, respectively. The deterministic time–frequency analysis and the deep learning method are recognized as the standard for objective wheeze detection in other studies on digital stethoscope recordings [32,40,41,42,48]. We aim to benchmark the two established concepts with the PIV mel spectrogram collected from the accelerometer patch for accuracy, sensitivity, and specificity to determine the optimal approach for wheeze detection.

The time–frequency method was used for each mel spectrogram to find the dominant frequency bands louder than the constant sound level, which represented wheezing, especially if the frequency band was greater than 100 Hz. In addition, the frequency band variation must be within 50 Hz, and the band duration must be longer than 100 ms, as shown in the flow chart in Figure 5c.i [32,43]. The time–frequency approach accurately extracted wheezing 87.45% of the time when compared with the study physician’s labeling with 81.39% sensitivity and 92.53% specificity. In addition, the time–frequency analysis showed that wheezing from the PIVs was consistently captured by the accelerometer patch and consistently exhibited frequency content similar to that captured with the digital stethoscopes. In addition, since the PIVs were bandpass-filtered, as shown in Figure 5a, signals like heartbeats or chest wall movement did not interfere as much with the deterministic time–frequency detection method due to their low-frequency content. A potential root for inaccuracy in the time–frequency method is that wheezing frequency variation may exceed 50 Hz, especially during the beginning or end of a patient’s respiration cycle [43]. However, this criterion is necessary to reduce the impact of white noise interference; ignoring this criterion would increase the false positive rate of the wheeze detection and lower its specificity. Therefore, a more robust computational detection method, such as a deep neural network, is desirable.

**Figure 6:**
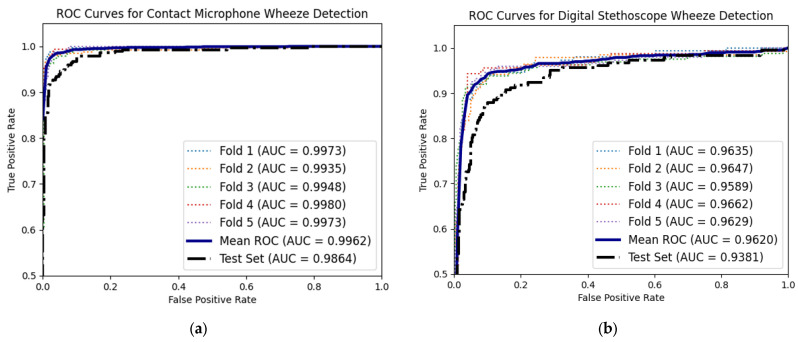
Area under the curves (AUC) of the receiving operating characteristics (ROC) of all five cross-validation folds of 2D convolutional neural network for wheeze detection with (**a**) accelerometer patch mel spectrograms and (**b**) digital stethoscope mel spectrograms, along with each respective mean AUC of the ROC shown in solid blue line. Test set of each respective AUC of ROC is also shown with dashed black line.

One popular deep learning model for image classification is CNN [40,41,50,51,52]. Since the captured PIVs were represented as mel spectrogram images, the accelerometer patch mel spectrograms can be used as inputs for this deep learning model. Due to the wide variety of patient data collected and the robustness of the sensor, no data augmentation was necessary. The dataset was initially partitioned with a dedicated test set to provide a more substantial gauge of the predictive model’s generalizability in real-world scenarios. Additionally, a five-fold cross-validation was also utilized on the remaining dataset to reduce overfitting and for hyperparameter optimization. In the 2D CNN model, convolutional layers were utilized to extract features of the mel spectrogram where the (3,3) kernel size allowed for the model to extract the wheezing frequency variation in the 64-band mel spectrograms. Dropout and max pooling were also implemented in tandem with the convolutional layers to improve generalization performance and reduce overfitting. In addition, ReLU activation function was utilized to provide non-linearity, which allowed the model to learn the curvature of the wheezing frequency variation, which was captured and shown in mel spectrogram—for example, in Figure 5b. The deep learning model folds consistently outperformed the deterministic time–frequency method in wheezing classification, with an average accuracy, sensitivity, specificity rate of 94.59%, 95.82%, and 93.41% over the five-fold cross validation. The model’s average AUC of the ROC and PR of the 2D CNN model was 0.9969 and 0.9842, respectively. On the test set, the deep learning model maintained its superior performance over the deterministic method with an accuracy, sensitivity, and specificity of 94.52%, 93.45%, and 96.16%, respectively, showing its ability generalizability in real-world scenarios. The AUC of the ROC and PR on the test set was 0.9864 and 0.9852, respectively, which shows the quality of the binary deep learning classifier. A comparison of the deterministic method and the deep learning classifier on the test set is shown in Table 3, with the AUC of the ROC curve of the five folds and the test set shown in Figure 6a.

### 3.3. Digital Stethoscope Computational Wheeze-Detection Method Performance

Both computational wheeze detection methods were also tested on the same patient set with the data captured from the digital stethoscope. The computational methods were tested on the digital stethoscope data to determine whether the accelerometer patch or digital stethoscope provided better data quality for computational wheeze-detection methods. The digital stethoscope’s time–frequency analysis wheeze detection on this patient set had an average accuracy, sensitivity, and specificity of 86.83%, 88.98%, and 84.52% over the five-fold cross-validation. The digital stethoscope deep learning model also outperformed the digital stethoscope deterministic method, with an average accuracy, sensitivity, and specificity rate of 89.54%, 92.34%, and 84.24%, respectively. The model’s average AUC of the ROC and PR of the 2D CNN model were 0.962 and 0.9274, respectively. On the test set, the digital stethoscope deep learning classifier showed similar performance with an accuracy, sensitivity, and specificity of 88.95%, 91.36%, and 86.65%, respectively, with AUC of the ROC and PR of 0.9381 and 0.8886. The AUC of the ROC curve of the five folds and the test set is shown in Figure 6b. The accelerometer patch wheeze-detection methods surpassed the digital stethoscope computational detection method performance, as shown in Table 3, which compares accuracy, sensitivity, and specificity. The computational methods performed worse on the digital stethoscope data in the detection and classification of wheezing or no wheezing due to increased noise in its data, which was from other people’s voices and ambient sounds in the mel spectrograms. The accelerometer patch’s superior data quality, unaffected by ambient sounds such as the accelerometer, only captures the patient’s PIVs, resulting in fewer false positives compared to the digital stethoscope data.

### 3.4. Patient Data

Both models were computed from a wide variety of patient data captured from the accelerometer patch to provide robustness for real-world applications and to objectively determine which computational wheeze-detection method had higher performance. Data included patients who had overlapping respiratory diseases, patients with high BMI, patients who were recently discharged from the hospital, and patients recorded in noisy environments.

**Figure 7:**
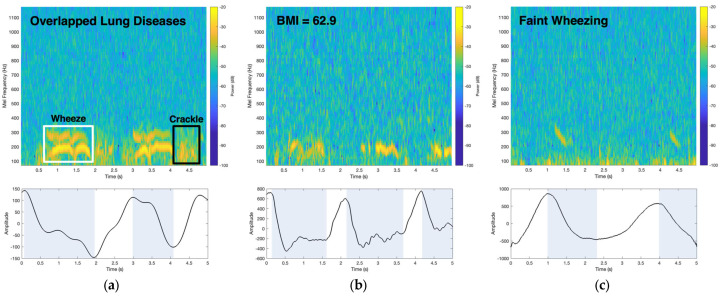
(**a**) Accelerometer patch mel spectrogram of a patient with asthma overlapped with COPD from the posterior right lower lobe containing wheezing, as shown with the thick bands encompassed within the white box, along with crackles, as shown with the higher sound amplitudes encompassed within the black box. In the accelerometer amplitude plot, wheezing occurred during expiration, as highlighted by the blue boxes. Crackles in the mel spectrograms occurred after wheezing and were estimated to occur during early inspiratory respiration phase. (**b**) Accelerometer patch mel spectrogram shows wheezing with the thick bands captured from the left lower back of a patient with a BMI of 62.9. Wheezing coincided with expiration respiration phase, as shown in the amplitude plots highlighted by the blue boxes. (**c**) Accelerometer patch mel spectrogram shows faint wheezing from a recently discharged patient, matched with expiration. All audio recordings are provided in the Supplementary Materials.

To accumulate the diverse dataset, the accelerometer patch was tested on patients with overlapping respiratory diseases, where other adventitious lung sounds may occur along with wheezing. One example is patient 34, who had a BMI of 29.5 and was tested on each of the nine auscultation sites in the hospital emergency room. As shown in the mel spectrogram of Figure 7a, this patient had monophonic wheezing, represented by the thick harmonic yellow bands in the white box, as adventitious lung sounds are louder than breath sounds [21]. After the wheezing occurred in the mel spectrogram, crackles were also captured, as shown by the patchy, non-harmonious, brighter yellow hue, with an example encompassed within the black box of the mel spectrogram. Crackle is another adventitious respiratory sound, which sounds like a rapid succession of crackling sounds. This recording containing both adventitious respiratory sounds was captured from the posterior right lower lobe of the lung, where the estimated expiration respiratory phase was highlighted by the blue box of the chest wall amplitude plot in Figure 7a. By analyzing the mel spectrogram along with the coincided respiration phase, this patient was determined to have expiratory monophonic wheezing with early inspiratory crackles following afterward. Expiratory wheezing is a known marker for intrathoracic airway obstruction, as seen in asthma, while the early inspiratory crackles could be another strong indicator of COPD, as there have been studies that correlated early inspiratory crackles with COPD, especially since this accelerometer patch mel spectrogram recording was taken from the patient’s lower lobes [53]. Audio playback of both the accelerometer patch and digital stethoscope recordings to the physician agreed with the notion of expiratory wheezing along with coarse early inspiratory crackles. The patient was characterized and diagnosed with asthma-COPD overlap syndrome (ACOS), which depicts an asthmatic patient who has developed chronic lung damage manifesting as COPD determined by lung function testing.

The accelerometer patch’s ability to capture PIVs from patients with obesity (BMI > 30) was also examined, as patients with obesity have a higher risk of asthma and COPD [54]. Patient 33 had a very high BMI of 62.9, with the examination performed in the hospital setting (emergency room). For obese patients, the accelerometer patch was placed and secured with a medical tape with no external pressure applied, while for typical auscultation examinations, physicians apply direct pressure to their stethoscope against the body to be able to listen to the acoustics of the lung. For the majority of obese people, auscultation sites correlating to the posterior lower lung lobes contain the highest chest wall fat content. Even when the accelerometer patch was placed on these sites to evaluate their posterior lower lobes, the device was still able to clearly capture the PIVs of the monophonic wheezes from the lung, as the patient’s mel spectrogram had thick bands representing the monophonic wheezing, as shown in the mel spectrogram in Figure 7b. In addition, the amplitude plot of Figure 7b shows the estimated expiration determined from the patient’s chest wall movement, as highlighted by the blue boxes. By analyzing the mel spectrogram and the coincided respiration phase, monophonic wheezing was determined to occur during the patient’s expiration. Audio playback of the accelerometer patch and digital stethoscope recordings to the physician confirmed the recording to be monophonic wheezing. Clinically, this patient was diagnosed with moderately persistent asthma.

The accelerometer patch was also tested on a patient who was recently hospitalized with acute asthma and was recently discharged at the time of testing. Even though the patient, patient 35, with a BMI of 20.7, was discharged, the patient still exhibited faint monophonic wheezing according to the physician’s auscultation with the traditional stethoscope, as shown in Table 2. The accelerometer patch was able to capture faint wheezing from the posterior lower left lobe, as shown in Figure 7c, where faint wheezes were captured and illustrated. The color scale amplitude of the thick band in the mel spectrogram is not as high relatively compared to other patients’ wheezing mel spectrograms. The patient’s faint wheezing was also found to occur during expiration.

### 3.5. Comparison of Accelerometer Patch and Digital Stethoscope in a Noisy Environment

Since respiratory wheezing occurs due to airflow limitations in the airways, multiple auscultation sites can capture the same high-pitched sounds. Therefore, to benchmark the accelerometer patch with the digital stethoscope, more specifically in a noisy hospital environment, simultaneous testing of the two sensors was performed in the emergency room, where other voices and machine noises are prevalent, as hospitals are a known source of high noise pollution [55,56]. Since we were testing patients in the hospital emergency room, the testing protocol allowed for the accelerometer patch and digital stethoscope to simultaneously capture data from the same breath sounds, although placed in different auscultation zones.

**Figure 8:**
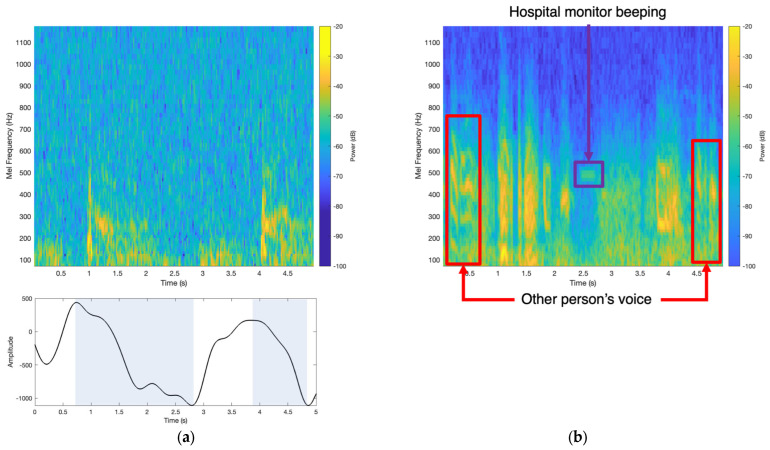
(**a**) Simultaneously recordings were performed on a patient in the emergency room with the accelerometer patch placed on the anterior left upper lobe, which captured monophonic wheezing, as shown by the mel spectrogram’s thick bands. The amplitude plot showed that the wheezing occurred during expiration, as highlighted by the blue boxes, while (**b**) the digital stethoscope captured monophonic wheezing from the anterior right upper lobe of the lungs. Noise artifacts, such as other people’s voices and hospital machines, were also present in digital stethoscope recordings and mel spectrograms. Both audio recordings are provided in the Supplementary Materials.

In this example, the accelerometer patch was placed across the anterior upper right lobe zone, and the digital stethoscope was placed in the anterior upper left lobe zone on patient 32, who had a BMI of 39.26. As shown in the mel spectrogram of Figure 8a, the accelerometer patch captured thick bands in the mel spectrogram, which represent the monophonic wheezing. The amplitude plot of Figure 8a shows the estimated expiration determined from the patient’s chest wall movement, as highlighted by the blue boxes. Therefore, by analyzing the mel spectrogram along with the coincided estimated respiration phases, this patient was estimated to have monophonic wheezing during expiration. However, the digital stethoscope mel spectrogram showed not only the thick bands related to the monophonic wheezing but also artifact noises, such as human voices and the hospital monitor device alarm, which were present in the background, as shown in Figure 8b, which agreed with the physicians’ notes based on sound playback. More specifically, the digital stethoscope mel spectrogram showed that the voices and other artifact noises that were picked up were in the similar frequency range of wheezes with similar attributes, which may lead to uncertainty in the interpretability of the signal along with the source of the signal in a time–frequency approach or a deep learning model, thus leading to more false positives in both computational wheeze detection performance. Therefore, the accelerometer patch is ideal for robust automatic detection of wheezing in all environments, especially noisy ones, as it only captures the PIVs of the lungs. This patient was diagnosed with ACOS. All the audio files of the accelerometer patch and digital stethoscope recordings are provided in the Supplementary Materials.

## 4. Discussion

Utilizing objective computational wheeze-detection methods with a low-profile wearable accelerometer patch that is insensitive to ambient sounds can provide patients at risk of respiratory disorders like asthma with a remote and extended monitoring tool that can be used for pre-emptive, in-depth longitudinal analysis of the respiratory status of their lungs.

Both computational wheeze-detection methods using the accelerometer patch data were shown to have a high accuracy rate when using the diverse, real-world patient dataset. The time–frequency analysis method was utilized to detect wheezing automatically and to also show that the wheezes captured from the accelerometer patch share similar characteristics as wheezes captured from the digital stethoscope. In comparison, the deep learning 2D CNN model integrated with the accelerometer patch showed that it could be used to detect wheezing from the adult population with a higher accuracy, sensitivity, and specificity rate compared to the time–frequency approach. In addition, these computational wheeze-detection methods also outperformed when utilizing accelerometer patch data compared to digital stethoscope data when captured on the same patient set, as shown in Table 3, as the accelerometer patch only captured the patient’s PIVs. This shows the robustness of the accelerometer patch and deep learning model system.

Individuals with asthma, COPD, and other respiratory disorders exhibit diverse demographic profiles, with notable variations observed, particularly in relation to obesity, as obesity is an important risk factor for airway obstruction diseases [54]. The accelerometer patch excels in capturing PIVs, showcasing its effectiveness even in scenarios where fat content on extremely obese people poses a potential obstacle for sound transmission. Moreover, the inclusion of data on individuals with ACOS in the algorithm becomes crucial, as wheezing and crackles serve as vital features that can offer deeper insights for physicians [57]. Both of these features have been shown to be captured by the accelerometer patch, as shown in Figure 7a and Figure 7b, respectively.

By collecting the PIVs from a wide range of patients, including those with extreme levels of obesity and those with overlapping adventurous lung sounds, the application of the accelerometer patch and deep learning model framework, which integrates the collected data, is fortified as a valuable tool for detecting wheezing in individuals with varying fat content. To further enhance the deep learning model framework, additional data collection from patients with other adventitious lung sounds would provide more distinctions. To strengthen the deep learning model, gathering diverse lung sound data can enhance its ability to identify wheezing in individuals at risk of asthma or COPD.

These wheeze-detection findings were also shown to be robust, as the accelerometer patch collected data on patients in vastly different environments, especially in the hospital emergency room, where patient monitoring is essential. The accelerometer patch has also shown high fidelity in capturing lung wheezing, especially as the accelerometer patch is not sensitive to ambient sounds, as shown in Figure 8a. This robustness is also illustrated by the high performance of the accelerometer patch computational wheeze-detection method. By utilizing the full extent of the accelerometer, not only can the PIVs be used to automatically detect and characterize wheezes, but also extract the macro body movements, such as respiratory rate/phase, to determine additional key features for respiratory care, such as if the wheezing is inspiratory or expiratory and the patient’s chest wall amplitude range, to help with respiratory disease diagnosis. Additionally, since the accelerometer patch can capture faint wheezing, especially in patients who were recently discharged from the hospital, as shown in Figure 7c, this framework could be deployed via telehealth as the accelerometer patch can be used longitudinally and has a small form factor. Moreover, the objective, automatic methods can provide a robust approach to detecting respiratory wheezing in patients.

Furthermore, the accelerometer patch algorithm can be adapted to detect various adventitious lung sounds and respiratory indicators, such as crackles and coughing, associated with diseases like asthma and COPD [4,6,58,59,60]. Coughing, a natural defense mechanism, can be characterized by various sounds, such as wet or brassy [58,60,61,62,63]. Coughing consequently triggers significant chest wall movements, which can be distinctly captured by an accelerometer [64]. Further data collection and physician labeling can enhance the algorithm’s capability to identify these additional respiratory signals.

## 5. Conclusions

This work builds toward the goal of an automatic, accurate, and objective means for monitoring and detecting wheezing by using a robust, wearable accelerometer patch. This accelerometer patch allows for the PIVs of wheezing from a multitude of patients with varying BMIs, respiratory disorders, and at different stages of their treatment to be captured and detected with the use of deep learning. The deep learning classifier has exhibited great performance, surpassing the deterministic time–frequency classifier in terms of accuracy, sensitivity, and specificity, achieving rates of 94.52%, 93.45%, and 96.16%, respectively, on the test data set. Additionally, the AUC of ROC and PR curves has a value of 0.9864 and 0.9852, respectively, which further validates the efficacy of the binary classifier. Furthermore, the computational wheeze-detection methods demonstrated superior performance when using data captured from the accelerometer patch compared to the digital stethoscope data collected from the same set of patients. The respiratory phase and the chest wall movement can also be extracted by the accelerometer and used as indicators of asthma and COPD. As a result, this framework, combining a wearable accelerometer patch and deep learning, holds potential for remote patient monitoring and integration within telehealth platforms. Providing a non-invasive, rapid, and cost-effective analysis of a patient’s respiratory and lung status can help physicians deliver more precise and expedited treatment to their patients. Future directions include testing more patients with different adventitious lung sounds with the accelerometer patch to capture different PIVs, such as crackles and decreased breath sounds. This broader dataset will bolster the deep learning classifier framework, enabling the classification of various abnormal lung sounds.

## Figures and Tables

**Table 1:** Patient characteristics of 52 patients tested at Grady Memorial Hospital.

Patient Characteristics	Values
Gender (Male/Female)	23/29
Emergency Room/In Clinic	34/18
Age: mean (standard deviation: SD), range	56.74 (13.63), 33–87
Height (m): mean (SD), range	1.71 (0.11), 1.47–1.96
Weight (kg): mean (SD), range	96.32 (35.41), 55.3–215
BMI: mean (SD), range	31.76 (9.14), 18.55–62.9

**Table 2:** Detailed description of each patient tested, along with lung auscultation notes, diagnosis provided by physician, and where they were tested.

Patient #	Age (Years Old)	Sex	Height (m)	Weight (kg)	BMI	Lung Auscultation Notes	Diagnosis	Testing Location
1	53	M	182.9	83	24.8	Polyphonic Wheezing	Severe Asthma	Clinic
2	50	M	195.6	112.9	29.5	No Wheezing	Mild Asthma	Clinic
3	61	F	152.3	77.6	33.4	No Wheezing	ACOS	Clinic
4	60	M	182.2	85.3	25.5	No Wheezing	Mild Asthma	Clinic
5	67	M	185.4	108	31.4	No Wheezing	Severe Asthma	Clinic
6	52	F	157.5	55.8	22.5	Wheezing	Severe Asthma	Clinic
7	45	F	172.7	97.5	32.7	No Wheezing	Mild Asthma	Clinic
8	63	F	167.6	102.1	36.3	No Wheezing	Mild Asthma	Clinic
9	53	F	170.2	126.1	43.5	Wheezing	Severe Asthma	Clinic
10	36	F	157.5	109.3	44.1	No Wheezing	Moderate Asthma	Clinic
11	58	F	147.3	83.9	38.7	Wheezing	Severe Asthma	Clinic
12	50	F	167.6	106.6	37.9	No Wheezing	Chronic Hives	Clinic
13	70	M	185.4	66.2	19.3	Wheezing	Severe ACOS	Clinic
14	39	F	172.7	65.8	22	Wheezing	Severe Asthma	Clinic
15	57	F	165.1	78.5	28.8	Wheezing	Severe Asthma	Clinic
16	75	F	178	87.3	27.62	No Wheezing	Acute Respiratory Failure	Emergency Room
17	50	F	165	124.7	46.1	No Wheezing	Acute Respiratory Failure	Emergency Room
18	75	F	155	71.2	29.6	No Wheezing	COPD Exacerbation	Emergency Room
19	56	M	185	98.4	28.8	No Wheezing	ADHF	Emergency Room
20	58	M	172	55.3	18.55	No Wheezing	COPD Exacerbation	Emergency Room
21	79	F	168	90	32.12	No Wheezing	ESRD	Emergency Room
22	38	F	170	210	32.89	No Wheezing	ADHF	Emergency Room
23	67	F	165	215	35.84	No Wheezing	COPD Exacerbation	Emergency Room
24	55	F	157.5	79.4	31.2	Wheezing	Severe Asthma	Clinic
25	63	M	170.2	62.6	21.6	No Wheezing	Bilateral Pleural Effusion	Emergency Room
26	33	M	167.6	93	33.1	No Wheezing	CAP	Emergency Room
27	62	F	170.8	106.1	36.6	Wheezing	Moderate Asthma	Clinic
28	57	F	165.1	80.3	29.5	Wheezing	Severe Asthma	Clinic
29	36	M	188	98.4	27.9	Polyphonic Wheezing	Severe Asthma	Emergency Room
30	60	M	175.3	100.7	32.8	No Wheezing	COPD Exacerbation	Emergency Room
31	43	F	167.5	150.6	53.6	No Wheezing	Sarcoid	Emergency Room
32	87	F	152.7	91.2	39.26	Wheezing and Crackle	Reactive Airway Disease	Emergency Room
33	35	F	160	161	62.9	Wheezing	Severe Asthma	Emergency Room
34	63	M	175.2	90.7	29.53	Wheezing and Crackle	Reactive Airway Disease	Emergency Room
35	43	F	175.6	63.5	20.7	Wheezing	Asthma Exacerbation	Emergency Room
36	35	M	165.1	72.6	26.63	No Wheezing	Pneumonia	Emergency Room
37	64	M	180.3	92.5	28.06	Wheezing	Acute Respiratory Failure	Emergency Room
38	77	M	177.8	76.7	24.36	Wheezing	COPD Exacerbation	Emergency Room
39	59	F	162.2	99.3	37.6	Wheezing	COPD Exacerbation	Emergency Room
40	72	M	162	59	22.5	Wheezing	COPD Exacerbation	Emergency Room
41	missing	F	167.7	63	22.4	Wheezing	Severe Asthma	Emergency Room
42	40	F	165.8	81.6	29.7	Wheezing	Acute Respiratory Failure	Emergency Room
43	56	M	180.3	120.2	37	Wheezing and Crackle	ADHF	Emergency Room
44	84	M	188	147.4	41.7	Wheezing	ADHF + CAP	Emergency Room
45	67	F	170	89.4	37.2	Wheezing and Crackle	COPD Exacerbation	Emergency Room
46	65	F	167.6	44.9	16	Wheezing and Crackle	COPD Exacerbation	Emergency Room
47	72	M	178	100.2	31.7	Wheezing and Crackle	COPD Exacerbation	Emergency Room
48	80	M	180	83	25.5	Wheezing	Right Upper Lobe Mass	Emergency Room
49	49	M	185.4	91.2	26.5	Wheezing and Crackle	Acute Respiratory Failure	Emergency Room
50	43	M	190.5	68	18.7	Wheezing	Severe Asthma	Emergency Room
51	27	M	175.3	74.8	24.3	Wheezing	Severe Asthma	Emergency Room
52	55	F	154.9	123.9	51.6	Wheezing	Severe Asthma	Emergency Room

Asthma–Chronic Obstructive Pulmonary Overlapped Syndrome is denoted as ACOS. Chronic Obstructive Pulmonary Disorder is denoted as COPD. Acute Decompensated Heart Failure is denoted as ADHF. Community-Acquired Pneumonia is denoted as CAP.

**Table 3:** Comparison of deterministic time–frequency detection and deep learning classifier tested on test data from accelerometer patch PIVs and digital stethoscope recordings.

Detection Method	Accuracy (%)	Sensitivity (%)	Specificity (%)
Accelerometer Patch Time–Frequency Analysis	87.45	81.39	92.53
Accelerometer Patch 2D CNN on Test Set	94.52	93.45	95.72
Digital Stethoscope Time–Frequency Analysis	86.83	82.98	87.52
Digital Stethoscope 2D CNN on Test Set	88.95	91.36	86.65

## Data Availability

The data presented in this study are available on request from the corresponding author.

## Supplementary Materials

The following supporting information can be downloaded at: https://www.mdpi.com/article/10.3390/bios14030118/s1, Audio S1: ACM_Patient32_Loc2.wav, Audio S2: ACM_Patient33_Loc9.wav, Audio S3: ACM_Patient34_Loc4.wav, Audio S4: ACM_Patient35_Loc7.wav, Audio S5: DigitalStethoscope_Patient32_Loc1.wav.
